# Spiritual and religious perspectives in persons with visual impairment due to age-related macular degeneration

**DOI:** 10.3389/fpsyg.2023.1096215

**Published:** 2023-04-26

**Authors:** Carina Salzer, Lacramioara Samoila, Hosnasadat Mortazavi Moshkenani, Ovidiu Samoila

**Affiliations:** ^1^Department of Ophthalmology, Iuliu Hatieganu University of Medicine and Pharmacy, Cluj-Napoca, Romania; ^2^Vedis Ophthalmology Clinic, Cluj-Napoca, Romania

**Keywords:** age-related macular degeneration, religion, spirituality, depression, vision, elderly, coping

## Abstract

**Introduction:**

Age-related macular degeneration (AMD) is one of the global leading causes of severe vision loss. Patients suffering from AMD face complex spiritual and mental challenges that have an impact on the course of their disease, their quality of life, and their relationship with their surroundings.

**Methods:**

A survey was carried out using a 21-item questionnaire between August 2020 and June 2021 among 117 patients from different countries to investigate how spirituality, religion, and their way of practicing them affected the experiences and daily lives of patients suffering from AMD, and whether it helped them cope with the disease.

**Results:**

Our study concluded that spirituality and religion are important factors that facilitate patients’ ability to cope with a progressive degenerative disease such as AMD. Patients who are religious are more at peace with having AMD. Practices that contribute to patients being at peace in accepting the disease are regular prayers or meditation. Spirituality and religion are important components that promote a healthier and happier emotional state and mental wellbeing. In particular, by believing that death is not the end, patients feel more hopeful, which helps in their adjustment to a seemingly hopeless health condition. A significant number of AMD patients desire to talk about God with the medical staff. The profile of such patients could be those believing in a higher power, praying often, participating in religious services, being worried about the loss of vision, and needing assistance in daily life.

**Discussion:**

An interdisciplinary and multidimensional team of medical health professionals including mental health workers and chaplains can be of great value in managing persons with AMD.

## 1. Introduction

According to the World Health Organization (2022), age-related macular degeneration (AMD) is one of the global leading causes of severe vision loss. In 2019, nearly 67 million people were suffering from severe AMD, and the number is estimated to rise by 15% by 2050 (Li J. Q. et al., 2020). Additionally, managing the neovascular form of AMD is further increasing the costs in the healthcare system (Kim et al., 2019). The progressive degenerative disease of the eye has no cure and is often diagnosed late, thereby diminishing the quality of life by restricting patients’ ability to read, drive, perform daily tasks, and recognize faces (De Sousa Peixoto et al., 2021). Compared with patients suffering from glaucoma and diabetic retinopathy, studies show that the general psychological state is most impaired in people with AMD (Gusar et al., 2021).

Many AMD patients are at higher risk of developing depression out of fear of losing their vision even while the disease progresses to place higher restrictions on their daily activities (Rovner et al., 2009; Popescu et al., 2012).

Aging in itself can be challenging. Life-altering circumstances like retirement, the death of one’s spouse or friends, or reduced health capacity, can lead to a more restricted social network or potential loneliness thus affecting the emotional state of older adults (Alma et al., 2011; Yanguas et al., 2018). In comparison to healthy older adults, age-related visual impairment negatively affects the psychological state of the individuals and can even become a personal tragedy for some, especially when aging is accompanied by other limiting physical conditions (Wahl et al., 1999). The biggest challenge for some patients is having difficulties with self-care and home duties (Taylor et al., 2020).

Even mild vision loss impacts the emotional wellbeing of patients. Severe vision loss (visual acuity lower than 20/200, a frequent evolution of AMD) leads to negative psychological sequels in the later years, with limiting effects on behavioral competence as well as the ability to adapt emotionally (Finger et al., 2011). Given the popular belief that the eyes are the windows to the soul, sudden vision loss affects people not only from a physical point of view but also emotionally and mentally. This requires a good understanding from the physicians for implementing a holistic approach to the treatment that acknowledges the patients’ value system as well as their spiritual wellbeing to support better management of their overall health (Marzorati and Pravettoni, 2017).

There are internal and external resources that may help patients cope with the disease. These resources are identified in this study in terms of religion and spirituality. Though not always visible or expressed, religion and spirituality may help patients respond to ontological and soteriological questions and may change their perspective of the disease. For instance, AMD may be viewed as a “punishment” from God or a disease that helps the believer to reconnect with God in anticipation of the “end.” Responses to the disease are drawn from the already present (or absent) faith in an effort to find a sense of emotional comfort and connection (e.g., “God gives comfort and strength”), meaning (e.g., “the disease is a test”), control (e.g., “God can heal the disease”), or practical support (e.g., “help from church members”) (Sherman et al., 2021).

Religion and spirituality are conjoined concepts. While religion is seen more as an objective and external concept that is often practiced in an organized group setting, spirituality, on the other hand, is considered an internal, personalized, and divine encounter or unswerving connection with God (Hyman and Handal, 2006; Barber, 2012). Religion involves beliefs, practices, and rituals related to the sacred (God, deity, angels, ultimate truth) (Koenig, 2009) which are organized and practiced in a community.

According to Pargament (1997), religion is not just “one thing” as a coping mechanism but it is a force that can change forms at different times and places, assists in molding the coping process, and in return gets shaped in the process.

Spirituality, however, is more difficult to define. It is something people define for themselves that is largely free of the rules, regulations, and responsibilities associated with religion (Naghi et al., 2012). Spirituality may be linked to beliefs related to the same questions raised by religion (e.g., “Is there a Creator?” or “Is death the end?”), but the term may be extended beyond to include positive psychological, social, or character states.

Religion and spirituality may not only enhance mental health but also facilitate the physical wellbeing of patients as shown in a study conducted by Shattuck and Muehlenbein (2020), where physical parameters such as reduced blood pressure and inflammation were influenced by religiosity and spirituality. A study by Wang et al. (2008) on older adults in China with vision loss underlined the importance of spirituality and its positive effect on the adjustment process to age-related vision loss.

Religion helps in coping with stress by providing powerful cognition and an optimistic worldview as well as setting rules and regulations regarding the various aspects of life including the way of living, having compassion, how to treat others, etc. Thus, it makes support available for individuals in society when needed as well as reduces stress and increases positive emotions which leads to the enhancement of social and mental health. Although positive religious support is associated with reduced stress and depression, negative aspects of religion such as justifying hatred, prejudice, and aggression can increase depression, anxiety, and fear, and impair mental health (Koenig, 2012; Holt et al., 2018).

Religiously active individuals may have better therapeutic adherence due to a larger number of social contacts who encourage each other to seek medical guidance on time and to follow through with the prescribed medications (Koenig, 2004). In addition, a higher level of confidence in physicians as well as more trust in healthcare providers is found in religiously active individuals (Benjamins, 2006).

The primary objective of this study, therefore, is to investigate how AMD patients’ spirituality, religion, and their way of practicing them affect their experiences and daily lives, and whether it helps them in coping with the disease or not. One premise is that having AMD and a visual impairment increases patients’ interest in spiritual and religious topics. On the contrary, having AMD may challenge patients’ faith. One goal of the study is to assess a possible correlation between patients with AMD who believe in a higher power and their perspective on their disease, and patients who do not consider themselves religious or spiritual.

In addition, by assessing whether regular prayer or meditation had an ameliorating influence on patients dealing with such a degenerative eye condition, we intend to determine if prayer provided comfort and strength while experiencing fear of vision loss.

Our study also provides comparative data of Romanian and international patients (from nine countries) diagnosed with AMD obtained through a questionnaire.

## 2. Materials and methods

### 2.1. Design and instruments

A cross-sectional study was conducted through a 21-item questionnaire from August 2020 until June 2021. In this study, Romanian patients with AMD who visited the “Vedis” clinic as well as the online population of English-speaking and German-speaking patients with AMD were assessed.

The questionnaires were constructed based on previous works, namely the Magyar-Russel study regarding ophthalmology patients, and Büssing studies regarding the SpREUK scale and Spiritual Needs Questionnaire (Magyar-Russell et al., 2008; Büssing et al., 2014; Büssing, 2021). We did not compute questionnaire scores; rather individual questions were compared and correlated among participants. The main categories of the questionnaires were related to religious practices (church attendance/participation in religious services, faith, denominations, etc.), spiritual practices (meditation, prayer, etc.), existential identity (meaning of life, acknowledgment of the end, making peace with the disease, etc.), general emotional state (feeling happy, hopeful, sad, down, cared for, lonely, etc.) and relationships (getting help, support in daily activities, etc.). The response choices were categorical (yes/no) or ordinal (all the time/often/sometimes/rarely/never) among other options. The questionnaires could be completed in 10 min and were collected anonymously. Approval was obtained from the Ethics Committee of the Iuliu Haţieganu University of Medicine and Pharmacy Cluj-Napoca.

The data collection was carried out through a non-probable type of sampling using a multisampling method including a consecutive sampling method for the Romanian patients and a convenient sampling method for the international population. In Romania, the survey was administered to 98 consecutive AMD patients being treated for neovascular disease during their routine eye examination under the supervision of OS. The questionnaires were filled out anonymously by the patients before their appointment or could be taken home and then returned, or patients could have the assistance of an interviewer for answering the survey at the clinic. In the end, 48 responses to the surveys were collected and one was rejected due to the exclusion criteria (described below) and the final data included 47 Romanian respondents.

For the international respondents, a 24-item questionnaire in English and German was made available online on two different Facebook self-supporting groups with people having AMD. The questionnaire was the same as the Romanian one but with a few modifications; it included three additional questions on participants’ country of residence, type of macular degeneration, and stage of AMD.

The English questionnaire was available online from 8 to 12 October 2021 and 38 responses were collected of which one was rejected due to the exclusion criteria. The German questionnaire was accessible online from 13 to 19 October 2021 and 34 responses were submitted of which one had to be excluded due to the exclusion criteria.

### 2.2. Participants

A total of 117 respondents were included in the study. The majority of the respondents were women in the Romanian (57.4%, 27 patients out of 47) and the international (84.3%, 59 patients out of 70) group. In the international group, the age range was between 29 and 86 years, with an average of 64.3 ± 13.1 years, and in the Romanian group, it was between 31 and 87 years of age, with an average of 68.6 ± 11.8 years. Patients younger than 60 years were seen in both groups, 19.3% in the Romanian group, and 37.1% in the international group. The patients’ demographics and AMD characteristics (type, vision, duration, treatment) are presented in Table 1.

**TABLE 1:** Demographics of 117 patients and the duration and stage of AMD.

		Number (%) of Romanian patients	Number (%) of international patients
**Gender**			
	Female	27 (57.4%)	59 (84.3%)
	Male	20 (42.6%)	11 (15.7%)
**Age (years)**			
	21–30	0 (0%)	1 (1.4%)
	31–40	1 (2.1%)	3 (4.3%)
	41–50	2 (4.3%)	4 (5.7%)
	51–60	7 (14.9%)	18 (25.7%)
	61–70	15 (31.9%)	17 (24.3%)
	71–80	14 (29.8%)	18 (25.7%)
	81–90	8 (17%)	7 (10%)
	No answer	0 (0%)	2 (2.9%)
Age (years), mean ± SD		68.6 ± 11.8	64.3 ± 13.1
**Religions**			
	Christian	41 (87.2%)	35 (50%)
	Jewish	0 (0%)	1 (1.4%)
	Moslem	0 (0%)	0 (0%)
	Atheist	2 (4.3%)	6 (8.6%)
	Somebody who thinks there might be a God, but is not sure	2 (4.3%)	10 (14.3%)
	Somebody who thinks that it is not possible to know if there is a God	0 (0%)	7 (10%)
	Other	1 (2.1%)	9 (12.9%)
	No answer	1 (2.1%)	2 (2.8%)
**Vision**			
	Poor	18 (38.3%)	17 (24.6%)
	Fair	22 (46.8%)	30 (43.5%)
	Good	7 (14.9%)	22 (31.9%)
	No answer	0 (0.0%)	1 (1.4%)
**Duration of AMD**			
	0–3 years	24 (51.1%)	29 (41.4%)
	3–6 years	11 (23.4%)	16 (22.9%)
	6–9 years	2 (4.3%)	9 (12.9%)
	9–12 years	2 (4.3%)	5 (7.1%)
	> 12 years	2 (4.3%)	7 (10%)
	No answer	6 (12.8%)	4 (5.7%)
Duration of AMD (years), mean ± SD		4.7 ± 3.4	5.8 ± 6.8
**Intraocular injection**			
	Received	36 (76.6%)	53 (75.5%)
	Not received	10 (21.3%)	17 (24.3%)
	No answer	1 (2.1%)	0 (0%)
**Type of AMD**			
	Dry	–	17 (24.3%)
	Wet	47 (100%)	50 (71.4%)
	I don’t know	–	2 (2.9%)
	No answer	–	1 (1.4%)
**Stages of AMD**			
	Early	–	17 (24.3%)
	Intermediate	–	26 (37.1%)
	Late	47 (100%)	12 (17.1%)
	I don’t know	–	15 (21.4%)
	No answer	–	0 (0.0%)

### 2.3. Inclusion and exclusion criteria

For the Romanian survey, we included patients who had a neovascular form of AMD, which was confirmed by fundoscopic eye examinations and ocular coherence tomographies (SD-OCT). All selected patients were scheduled for anti-VEGF intraocular injections and gave their consent for participation in this study.

For the online international survey, patients at any stage of AMD were included. In this group, both dry and wet AMD patients were involved, and there was no possibility to ascertain the accuracy of their diagnosis.

Patients with juvenile macular degeneration (macular dystrophies) and responses that had less than 50% valid items filled out were excluded from the study. However, in the Romanian group, patients younger than 60 years of age were also included even when clinical characteristics for macular dystrophy were not met but the case was fully diagnosed as AMD.

### 2.4. Statistical analysis

The statistical analysis was performed on IBM SPSS Statistics 28, with a *p*-value set at 0.05. The diagrams and tables were made using Google Sheets and Microsoft Excel.

With an aim to explore the associations between ordinal and continuous variables, we conducted Spearman’s correlation on our data. We also performed the Wilcoxon Rank-Sum test (Mann–Whitney-U-test) and the Kruskal–Wallis test to assess ordinal and continuous variables according to the categorical variables. In addition, we analyzed the categorical/binary variables through the chi-square test and Fisher’s exact test. The latter was considered only for those variables in which more than 20% of the expected cell values were less than 5.

## 3. Results

**FIGURE 1:**
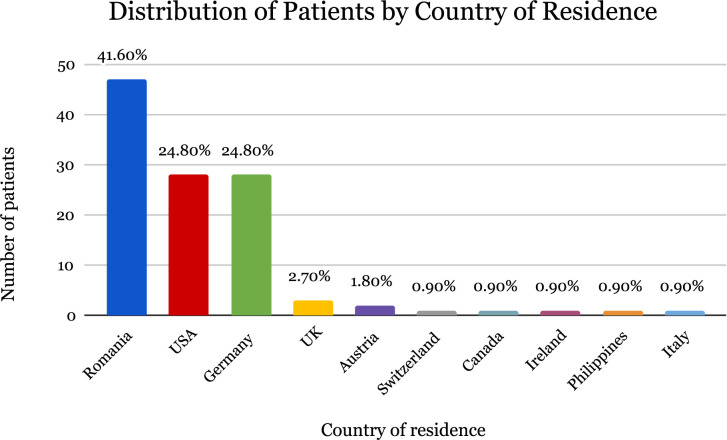
Country of residence.

In total, 117 patients were analyzed in two groups: the Romanian group with 47 patients, and the international group with 70 patients. The country distribution of the participants is presented in Figure 1.

While the majority of the Romanian patients (87.2%) considered themselves Christians, only half of the international group (50%) indicated Christianity as their religion. However, the international group was more diverse in terms of their religious beliefs than the Romanian group, with a higher prevalence of agnostics and atheists.

Regarding the level of vision, a higher percentage of international patients (31.9%) reported having good vision in comparison with Romanian patients (14.9%).

The average duration of AMD (time between first diagnosis to the date of questionnaire completion) in the Romanian group was 4.7 ± 3.4 years, and in the international group, it was 5.8 ± 6.8 years. The shortest duration of AMD was 1 day in the international group and 2 months in the Romanian group. The longest duration of AMD was 15 years in the Romanian group and 36 years in the international group.

Approximately 71.4% of the international patients self-declared having wet AMD, while the rest declared dry AMD. However, all the Romanian patients had wet AMD and were in the late stages of the disease. While 17.1% of the international patients self-declared to be in the late stages of AMD, 37.1% of these patients were suffering from the intermediate stage of AMD.

An overview of the questionnaire responses is presented in Table 2 (however, complex responses, related to the general emotional state of the patients, have not been presented).

**TABLE 2:** Overview of the responses from 117 patients.

		Number (%) of Romanian patients	Number (%) of international patients
**Worry about the disease**			
	Not worried	3 (6.4%)	2 (2.9%)
	Sometimes worried	12 (25.5%)	17 (24.3%)
	Worried	32 (68.1%)	51 (72.9%)
	No answer	0 (0%)	0 (0%)
**Being at peace with the disease**			
	Feeling outraged for having the disease	17 (36.2%)	28 (40%)
	Accepted the disease	29 (61.7%)	32 (45.7%)
	Feeling nothing toward the disease	0 (0%)	8 (11.4%)
	No answer	1 (2.1%)	2 (2.9%)
**Need of assistance**			
	No need for assistance	31 (66%)	53 (75.7%)
	Needs assistance	15 (31.9%)	17 (24.3%)
	No answer	1 (2.1%)	0 (0.0%)
**Source of assistance**			
	Family	15 (31.9%)	19 (27.1%)
	Friends	5 (10.6%)	1 (1.4%)
	People from work	0 (0%)	1 (1.4%)
	People from church, synagogue, or mosque	0 (0%)	0 (0%)
	Technologies	–	1 (1.4%)
	No answer	27 (57.5%)	48 (68.7%)
**Believe in a higher power**			
	No, I do not believe at all	5 (10.6%)	15 (21.4%)
	Partially believe in a higher power	10 (21.3%)	19 (27.1%)
	Strongly believe in higher power	31 (66%)	36 (51.4%)
	No answer	1 (2.1%)	0 (0.0%)
**Religious vs. non-religious**			
	Non-religious	8 (17%)	33 (47.1%)
	Religious	38 (80.9%)	37 (52.9%)
	No answer	1 (2.1%)	0 (0.0%)
**Participation in religious services**			
	Never	6 (12.8%)	27 (38.6%)
	Rarely	8 (17%)	15 (21.4%)
	Sometimes	16 (34%)	10 (14.3%)
	Often	16 (34%)	18 (25.7%)
	No answer	1 (2.2%)	0 (0.0%)
**Prayer or meditation frequency**			
	Never	4 (8.5%)	18 (25.7%)
	Sometimes	9 (19.1%)	27 (38.6%)
	Everyday	33 (70.2%)	25 (35.7%)
	No answer	1 (2.2%)	0 (0.0%)
**Comfort in prayer while fearing loss of vision**			
	Find comfort in prayer	9 (19.1%)	30 (42.9%)
	Sometimes find comfort in prayer	14 (29.8%)	21 (30%)
	Do not find comfort in prayer	24 (51.1%)	19 (27.1%)
	No answer	0 (0.0%)	0 (0.0%)
**God’s influence on the disease**			
	God comforts them in the disease	20 (42.6%)	19 (27.1%)
	God does not cause the disease, but he allows it	7 (14.9%)	16 (22.9%)
	God can heal the disease	14 (29.8%)	8 (11.4%)
	God has no influence on the disease	8 (17%)	14 (20%)
	The disease is a punishment	2 (4.3%)	2 (2.9%)
	The disease is a test	3 (6.4%)	4 (5.7%)
	God can make a blessing out of the disease	16 (34%)	13 (18.6%)
	No, because I do not believe	–	14 (20%)
	No answer	2 (4.3%)	2 (2.9%)
**Effect of AMD on faith**			
	Started struggling more in my spiritual walk	0 (0%)	3 (4.3%)
	It did not affect my spirituality	36 (76.6%)	54 (77.1%)
	Made me grow more spiritually and I find strength in my faith	10 (21.3%)	12 (17.1%)
	No answer	1 (2.1%)	1 (1.5%)
**Faith as help in daily life**			
	No, because I do not believe	4 (8.5%)	15 (21.4%)
	At the moment I struggle more with my faith	1 (2.1%)	9 (12.9%)
	Sometimes, I have moments where it helps me and moments where I struggle with my faith	10 (21.3%)	23 (32.9%)
	Faith gives me comfort and strength	32 (68.1%)	22 (31.4%)
	No answer	0 (0.0%)	1 (1.4%)
**Perspective on death**			
	Believe that death is the end	5 (10.6%)	9 (12.9%)
	Not sure about death being the end	17 (36.2%)	21 (30%)
	Believe that death is not the end	23 (48.9%)	39 (55.7%)
	No answer	2 (4.3%)	1 (1.4%)
**Through the disease more interested in spiritual topics**			
	Disagree	14 (29.8%)	43 (61.4%)
	Partially agree	18 (38.3%)	17 (24.3%)
	Agree	12 (25.5%)	10 (14.3%)
	No answer	3 (6.4%)	0 (0.0%)
**The wish to talk about God with the medical staff**			
	Do not want to talk about God with medical staff	21 (44.7%)	59 (84.3%)
	Want to talk about God with medical staff	21 (44.7%)	10 (14.3%)
	No answer	5 (10.6%)	1 (1.4%)

**FIGURE 2:**
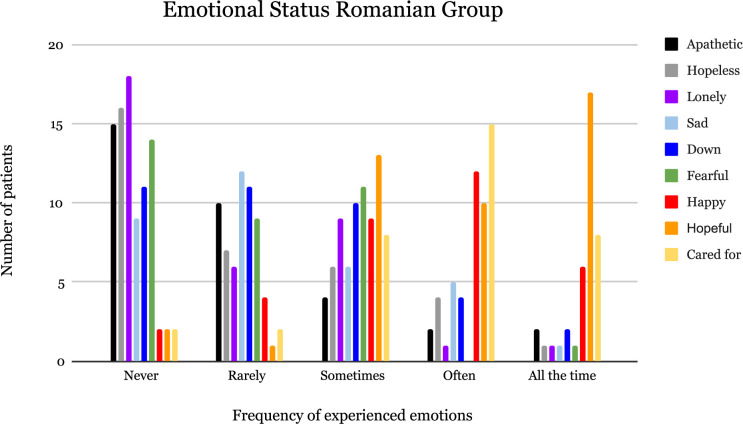
Emotional status of AMD patients from the Romanian group.

**FIGURE 3:**
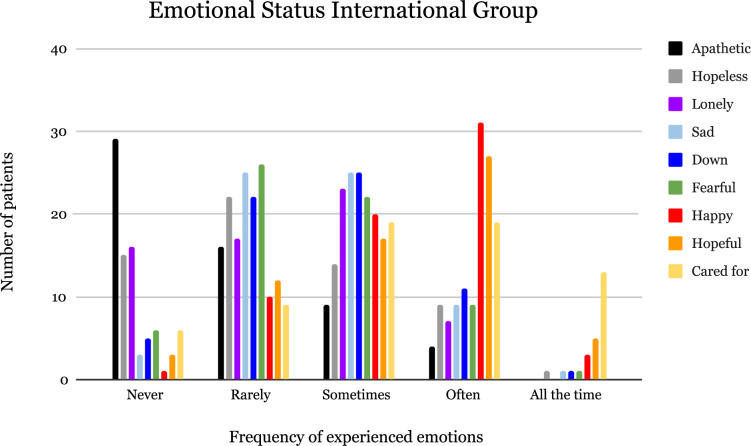
Emotional status of AMD patients from the international group.

The various emotions experienced by the AMD patients and the frequency of those emotions are shown in Figure 2 (Romanian group) and Figure 3 (international group). The majority of respondents from the international group reported often feeling happy in their lives and never apathetic; some even regarded feeling cared for all the time. In the Romanian group, the majority of patients described feeling happy, hopeful all the time, and never lonely, while some indicated often feeling cared for.

The international group had a higher score for “feeling down” compared to the Romanian group (*p* = 0.049). In general, the international group experienced positive emotions (happy, hopeful, and cared for) less often compared to the Romanian group.

The level of being worried about the disease was similar in both groups; the majority of patients in the Romanian group (68.1%) and the international group (72.9%) were worried about having AMD.

When it came to “being at peace with the disease”, the majority in both groups “accepted the disease” (45.7% among the international group and 61.7% among the Romanian group). However, 40% in the international group and 36.2% in the Romanian group were “feeling outraged for having the disease.”

A significant percentage of respondents in both groups reported that they needed assistance for carrying out their daily living tasks. However, this percentage was higher (but not statistically significant) in the international group (75.7%). The respondents from both the international and Romanian groups reported seeking assistance mainly from family members for their living tasks, but 10.6% of Romanian respondents also received assistance from friends.

The majority in both groups believed in a higher power; however, this percentage was higher among Romanian respondents (66% in the Romanian group vs. 51.4% in the international group, *p*-value not statistically relevant).

The international group consisted of a more equally distributed number of religious (52.9%) and non-religious (47.1%) patients than the Romanian group, where the highest number of patients (80.9%) considered themselves religious. Romanian patients were participating in religious activities more often than international patients. In addition, the percentage of international patients that never participated in religious activities was significantly higher (38.6%) than Romanian patients who never participated in religious activities (12.8%).

A higher percentage of Romanian patients prayed or meditated every day in their life (70.2%) than international patients (35.7%) though a higher percentage of international patients (42.9%) found comfort in prayer while fearing loss of vision than Romanian patients (19.1%).

On how God influences their disease, most patients from both groups believed that “God comforts them in the disease.” A significant percentage within the international group believed that “God does not cause the disease but allows it” (22.9%), “God has no influence on the disease” (20%), and “God can make a blessing out of the disease” (18.6%). Within the Romanian group, a significant percentage believed “God can make a blessing out of the disease” (34%) and “God can heal the disease” (29.8%).

The majority of patients in both international (77.1%) and Romanian (76.6%) groups reported that having AMD did not affect their spirituality and religiosity. However, a smaller percentage of international patients (14.3%) reported becoming more interested in spiritual and religious topics after their diagnosis, compared to Romanian patients (25.5%).

Many patients in both the international (31.4%) and the Romanian (68.1%) groups believed that their faith gave them comfort and strength to cope with the disease on a daily basis. A significant percentage of international patients (32.9%) believed that sometimes their faith helped them but at other times they struggled with it throughout the disease. In both international (55.7%) and Romanian (48.9%) groups, most patients believed that death was not the end.

In response to the question on whether patients would like to talk about God with the medical staff, 44.7% of the Romanian patients answered “yes,” 44.7% responded “no,” while 10.6% left this question unanswered. In contrast, most of the international patients (84.3%) did not want to talk about God with their health professionals.

The results showed that the poorer the vision of the person, the more likely they felt down in their life, according to a negative correlation (ρ = −0.392, *p* = 0.001) obtained from international patients with AMD. Similarly, among the Romanian patients, the poorer the vision, the more likely the person was worried about their condition (ρ = −0.495, *p* < 0.001).

Our study also demonstrated that patients from the international group who were at peace and accepted having the disease had a statistically significant higher mean of believing in a higher power (*p* < 0.01), finding comfort in prayer when they experienced fear of losing their vision (*p* = 0.002), participating in religious activities (*p* = 0.016), and praying or meditating on a regular basis (*p* = 0.010) than patients who were not at peace with the disease or did not feel anything toward the disease.

A correlation obtained from the data of the Romanian (*p* = 0.025) as well as the international group (*p* = 0.023) revealed that patients who are at peace with having AMD were more likely to believe that death was not the end. Besides, there was a correlation in both groups–Romanian (*p* = 0.013) and international (*p* < 0.001)–between “being at peace with the disease” and believing that “God can make a blessing out of the disease.” In addition, in the international group (*p* = 0.020), there was a link between patients who reported “being at peace with the disease,” and those who considered themselves religious.

Another finding of our study was the very strong correlation between gender and faith as a coping mechanism on an everyday basis (*p* = < 0.001) among the international respondents. Women, more than men, agreed that faith helped them on a daily basis by giving them “comfort and strength” or that sometimes they had “moments where their faith helps them and other moments where they struggle more with it.” Men agreed more with the statement that “at the moment they struggled more with their faith.” However, the international group had only a small number of men (15.7%). This gender difference was not found in the Romanian group, although the sample size contained more men than the international group. Regardless, a correlation (*p* = 0.002) was obtained between gender and being at peace with the disease, suggesting that women were more at peace in accepting AMD than men.

Patients from Romania who needed assistance in their daily activities had a statistically significantly higher mean in participation in religious activities (*p* = 0.044) as well as being worried about their eye condition (*p* = 0.048).

Patients who generally felt hopeless in their life had a statistically significant higher mean in need of assistance (*p* = 0.017), compared to those who did not need assistance in the international group. In Romania, patients who reported poorer vision had a statistically significant higher mean of receiving help from family and friends (*p* = 0.042). Interestingly enough, none of the patients indicated that they received help from their church, synagogue, or mosque members. This response option in the questionnaire was not selected by the international respondents either.

The results obtained in our study also revealed a negative correlation (ρ = −0.379, *p* = 0.027) between Romanian patients who believed in a higher power and a feeling of hopelessness and a negative correlation (ρ = −0.423, *p* = 0.011) between patients who generally felt cared for and participated in religious activities. Among international patients, we found a correlation (ρ = 0.251, *p* = 0.044) between generally feeling happy in life and finding comfort in prayer while fearing vision loss.

Our results also demonstrated that the international group of patients who were generally happy (*p* = 0.043) in their life had a statistically significant higher mean score in agreeing with the statement that “faith is helping them on an everyday basis to cope with the disease” by giving them comfort and strength in comparison with patients who answered “No, because I don’t believe,” “At the moment I struggle more with my faith,” and “Sometimes I have moments where it helps me and moments where I struggle with my faith.” The same applied to patients who generally felt hopeful (*p* = 0.010) within the international group.

We found an interesting observation when examining the correlation between happiness and religion, especially when comparing the differences between the countries. In our study, we compared Romania, Germany, and the USA and tried to identify in which country patients generally felt more cared for in their lives. We included only these three countries because the sample sizes from other countries were too small. According to our results, the AMD patients from the USA had a statistically significant higher mean of feeling generally cared for in their lives (*p* < 0.001) than patients from Romania and Germany, as patients from the latter were the ones who felt least cared for.

Our analyses also revealed that patients from the international group who felt generally happy in their life (*p* = 0.009) had a statistically significant higher mean in believing that God can make a blessing out of their disease, in response to whether “God has an influence on their health condition” in comparison with patients who did not choose this answer option. The results were similar for patients who generally felt hopeful in their lives (*p* = 0.007). This statistical significance could not be seen in the data acquired from the Romanian group.

On the other hand, data from the Romanian group showed that patients who generally felt down in their life (*p* = 0.027) had a higher statistically significant mean of believing the disease was a punishment than patients who did not choose this answer option. The results were similar for patients who generally felt hopeless in their lives (*p* = 0.029).

The results from the Romanian group also demonstrated that those who believed in a higher being (*p* = 0.020) had a statistically significant higher mean of believing that “God can heal the disease” in comparison with those who did not choose this answer. Likewise, those who participated in religious activities (*p* = < 0.001) had a statistically higher mean in believing that God could make a blessing out of the disease than patients who did not select this answer option.

Another association was found between being a religious person and becoming more interested in spiritual and religious topics with the onset of AMD. This applied to both Romanian (*p* = 0.014) as well as international (*p* = 0.017) patients. In addition, an association (*p* = 0.028) was established among Romanian patients who became more interested in spiritual and religious topics as a result of AMD and the desire to talk about God with the medical staff. There was also a strong link between Romanian patients who needed assistance in their daily life and their desire to talk about God with the medical staff (*p* = 0.014). Furthermore, in the international group (*p* = 0.031) as well as the Romanian group (*p* = 0.031), patients who believed in a higher being had a statistically higher mean to wish to talk about God with the medical team than patients who only partially believed or did not believe at all in a higher power.

### 3.1. Statistical analysis

The non-parametric statistical tests used for the evaluation of the variables are presented below. Results without significance were removed.

In Table 3 the results of the chi-squared and Fisher’s exact test for the Romanian group are presented. In Table 4 the results of the chi-squared and Fisher’s exact test for the international group are shared. Table 5 presents the Kruskal–Wallis test for the Romanian group, while Table 6 shares the results of the international group. In Table 7, the results of the Wilcoxon Rank-Sum test of the Romanian group are provided, while Table 8 contains the results of the international group. Finally, Spearman’s correlation test results for the Romanian and international groups are presented in Tables 9, 10, respectively.

**TABLE 3:** The results of the chi-squared and Fisher’s exact test for the Romanian group.

	*N*	Cramer’s V	Phi	*P*-value
Gender by being at peace with the disease	46	0.455	-0.455	0.002
Need of assistance by talk about God with medical staff	41	0.014	0.014	0.014
Being at peace with the disease by God can make a blessing out of the disease	44	0.375	0.375	0.013
Being at peace with the disease by believing death is not the end	44	0.409	0.409	0.025
Being a religious person by through the disease more interested in spiritual topics	43	0.450	0.450	0.014
Through the disease more interested in spiritual topics by talk about God with medical staff	39	0.441	0.441	0.028

**TABLE 4:** The results of the chi-square and Fisher’s exact tests for the international group.

	*N*	Cramer’s V	Phi	*P*-value
Gender by faith as coping mechanism on an everyday basis	69	0.537	0.537	<0.001
Being at peace with the disease by believing death is not the end	67	0.285	0.403	0.023
Being at peace with the disease by God can make a blessing out of the disease	66	0.434	0.434	<0.001
Being a religious person by through the disease more interested in spiritual topics	70	0.342	0.342	0.017
Being at peace with the disease by being a religious person	68	0.337	0.337	0.020

**TABLE 5:** The results of the Kruskal–Wallis test for the Romanian group.

		Mean	*P*-value	H-statistic	*N*	Mean rank
Worried about eye problem by faith as coping mechanism on an everyday basis			0.021	9.728		
	No, because I do not believe	1.75			4	7.63
	At the moment I struggle more with my faith	3.00			1	31.50
	Sometimes, I have moments where it helps me and moments	2.7			10	26.35
	Yes, it gives me comfort and strength	2.69			32	25.08
Believe in higher power by faith as coping mechanism on an everyday basis			< 0.001	25.992		
	No, because I do not believe	1.25			4	4.88
	At the moment I struggle more with my faith	2.00			1	10.50
	Sometimes, I have moments where it helps me and moments	0.7			10	15.15
	Yes, it gives me comfort and strength	2.91			31	29.02
Feeling fearful by through the disease more interested in spiritual topics			0.007	9.901		
	Disagree	1.27			11	10.36
	Partially agree	2.29			14	19.86
	Agree	2.44			9	22.56

**TABLE 6:** The results of the Kruskal–Wallis test for the international group.

		Mean	*P*-value	*H*-value	*N*	Mean rank
Believe in a higher power by being at peace with the disease			< 0.001	15.119		
	No	1.96			28	26.43
	I do not feel anything	1.89			8	26.44
Prayer or meditation by being at peace with the disease			0.010	9.193		
	No	1.86			28	27.54
	I do not feel anything	1.89			8	30.13
	Yes	2.38			32	41.69
Participation at religious services by being at peace with the disease			0.016	8.214		
	No	1.86			28	28.39
	I do not feel anything	1.88			8	28.06
	Yes	2.69			32	41.45
Comfort in prayer while fearing vision loss by being at peace with the disease			0.002	12.272		
	No	1.53			28	26.46
	I do not feel anything	1.62			8	29.63
	Yes	2.19			32	42.75
Feeling happy by faith as coping mechanism on an everyday basis			0.043	8.127		
	No, because I do not believe	3.08			13	26.15
	At the moment I struggle more with my faith	2.89			9	22.89
	Sometimes, I have moments where it helps me and moments	3.48			21	33.52
	Yes, it gives me comfort and strength	3.71			21	39.52
Feeling hopeful by faith as coping mechanism on an everyday basis			0.010	11.295		
	No, because I do not believe	2.85			13	25.00
	At the moment I struggle more with my faith	2.67			9	20.72
	Sometimes, I have moments where it helps me and moments	3.33			21	32.55
	Yes, it gives me comfort and strength	3.8			20	41.05

**TABLE 7:** The results of the Wilcoxon Rank-Sum test of the Romanian group.

		Mean	*P*-value	*Z*-value	*N*	Mean rank
Believe in a higher power by talking about God with medical staff			0.031	−2.153		
	No	2.29			21	17.64
	Yes	2.75			20	24.53
Worried about the eye problem by talk about God with the medical staff			0.036	−2.096		
	No	2.38			21	18.21
	Yes	2.81			21	24.79
Participation in religious services by need of assistance			0.044	−2.016		
	No	2.71			31	20.85
	Yes	3.33			15	28.97
Worried about the disease by need of assistance			0.048	−1.980		
	No	2.48			31	21.26
	Yes	2.86			15	28.13
Vision by where help is coming from			0.042	−2.034		
	Family	1.4			15	9.10
	Family and friends	2.22			5	14.70
Feeling down by the disease is a punishment			0.027	−2.216		
	No	2.21			34	17.59
	Yes	4.50			2	34.00
Feeling hopeless by the disease is a punishment			0.029	−2.182		
	No	1.87			31	16.13
	Yes	4.00			2	30.50
Believe in a higher power by God can heal the disease			0.020	−2.336		
	No	2.45			31	20.50
	Yes	2.93			14	28.54
Participation in religious services by God can make a blessing out of the disease			< 0.001	−3.891		
	No	0.76			29	17.62
	Yes	2.88			16	32.75

**TABLE 8:** The results of the Wilcoxon Rank-Sum test of the international group.

		Mean	*P*-value	*Z*-value	*N*	Mean rank
Believe in a higher power by talking about God with medical staff			0.031	−2.159		
	No	2.2			59	33.03
	Yes	2.8			10	46.6
Feeling hopeless by need of assistance			0.017	−2.398		
	No	2.12			45	27.88
	Yes	2.88			16	39.78
Feeling happy by God can make a blessing out of the disease			0.009	−2.618		
	No	3.24			50	29.14
	Yes	3.92			13	43.00
Feeling hopeful by God can make a blessing out of the disease			0.007	−2.721		
	No	3.12			50	28.6
	Yes	4.00			12	43.58

**TABLE 9:** The results of Spearman’s correlation for the Romanian group.

Variable 1	Variable 2	*N*	*p*-value	Correlation coefficient
Vision	Worried about the disease	47	< 0.001	−0.495
Believe in a higher power	Feeling hopeless	34	0.027	−0.379
Feeling cared for	Participation in religious services	35	0.011	−0.423

**TABLE 10:** The results of Spearman’s correlation for the international group.

Variable 1	Variable 2	*N*	*p*-value	Correlation coefficient
Felling down	Vision	63	0.001	−0.392
Feeling happy	Comfort in prayer while fearing vision loss	65	0.044	0.251
Feeling hopeful	Pray or meditation	64	0.033	0.267

In Table 11, the results of the Kruskal–Wallis test to determine which country the patients felt generally more cared for are presented. We included only data from patients from Romania, Germany, and the USA since the sample size from other countries were very small.

**TABLE 11:** The results of the Kruskal–Wallis test for Romania, Germany, and the USA.

		Mean	*P*-value	*H*-statistic	*N*	Mean rank
Feeling cared for by countries of residence			< 0.001	16.082		
	Germany	2.68			28	29.46
	Romania	3.71			35	50.83
	USA	3.88			26	53.88

## 4. Discussion

“Then will the eyes of the blind be opened and the ears of the deaf unstopped” (Isaiah 35:5)–a passage from the Old Testament, a sacred scripture for the Jews and Christians, where a renewed heaven and earth are described. Patients suffering from AMD face complex spiritual and mental challenges that have an impact on the course of their disease, their quality of life, and their relationship with their surroundings.

In our study we found that poor vision has an impact on patients’ mental health, leading them to feel generally down in their life, as seen in the international group. Poor vision also increases patients’ worry about AMD’s prognosis as evidenced in the Romanian group. Demmin and Silverstein (2020) arrived at a similar finding through their review of recent literature that patients with poor vision had a higher rate of depression and anxiety which affected their mental health. Similar findings by Casten and Rovner (2013) revealed that having AMD and the accompanying impaired vision caused depression in older adults. Loss of independence and reduction in quality of life are major factors that play a role in lowering the mood of people suffering from such eye conditions. Visual impairment in older adults has been linked to an increased risk of suicide among them (Waern et al., 2002).

Patients facing visual loss need multiple sources of support, including religion and spirituality (Yampolsky et al., 2008). A study done on 227 cancer patients from Lithuania who were hospitalized but were not terminally ill, showed that among spiritual needs, inner peace had the highest mean scores and that spiritual wellbeing had a positive effect on the emotional wellbeing of the patients contributing to life satisfaction and happiness (Riklikienė et al., 2020).

We found that believing “death is not the end” was associated with being at peace with having AMD. This finding was similar to the finding from a study done in the Detroit area in 1995 with 1,139 participants, which concluded that belief in eternal life as well as church attendance had a positive association with the wellbeing of the participants. Moreover, a strong belief in eternal life appeared to reduce the damaging effect of chronic health problems on psychological wellbeing but was unrelated to distress (Ellison et al., 2001).

In our study, being at peace with having AMD was associated with believing in a higher power among international patients. This peace was linked to praying or meditating on a regular basis and finding comfort in prayer when experiencing the fear of vision loss. Participating in religious activities also contributed to patients being at peace with the disease. Based on this, we assume that prayer and meditation, as well as participation in religious activities, are factors contributing to finding peace in acceptance of a progressive health condition. However, the same conclusion could not be made from data received from Romanian patients. Cultural differences in beliefs and practices could perhaps explain this variance between the international and Romanian patients.

Not just cultural but differences in gender were also interesting to examine. In the international group, while men struggled more with their faith when suffering from AMD, women agreed more that faith helped them on a daily basis, by providing them comfort and strength, although some agreed that they found themselves fluctuating between moments where their faith helped them, and then other moments when they struggled more with it. However, only a small number of men participated in the international group. This difference in responses between the genders was not found in the Romanian group, although it contained more men than in the international group. Meanwhile, an association was found between gender and being at peace with the disease, suggesting that women are more at peace with having AMD than men. This finding is similar to the findings of a study conducted by Büssing (2021) which concluded that women generally revealed higher spiritual needs than men. Likewise, this need is also higher in older adults.

A study done in Denmark, which is known to be a secular society, found that women were more religious and therefore used religious coping more than men. Women who experienced a crisis reported that it made them think more about religious questions than men; statistically, significantly more women (12.7%) than men (8.0%) perceived themselves as a stronger believer than before they experienced the crisis (Hvidtjørn et al., 2014). In Vienna, a study on cancer patients concluded that women lean to religion and spirituality more than men (Rassoulian et al., 2021). Reasons for this might be, as Li Y. I. et al. (2020) explained in their research, that women are more risk averse than men and religiousness can be seen as a type of risk aversion. On the contrary, Reid-Arndt et al. (2011) published a study on men and women with significant health conditions which showed that there was no difference between the genders regarding their self-reported level of religious practices and spiritual experiences thus indicating that both genders may benefit from the support of their beliefs as a coping mechanism for their disease.

Patients who generally felt more hopeless needed more assistance in their life, as seen in the international group. Losing their independence had an emotional impact on patients, who now had to depend on others due to their vision loss. Patients with poorer vision received help from family and friends in the Romanian group. Interestingly, support from the church, synagogue, or mosque was not chosen as a response option in either of the groups. Our finding is similar to the one in a study from the University of Jena in Germany which demonstrated that the greatest fear of patients regarding health and illness was “Being in need of help from others,” followed by the fear of having “an incurable disease.” The study also showed that participants expected that most help in life would come from “Family and Relatives” and “Medicine.” Religion and church ranked sixth among religious participants and were the least significant for non-religious participants (Keinki et al., 2022).

Garssen et al. (2015) discovered through their qualitative study on cancer patients in Netherlands, that none of their patients indicated that they received social support by attending religious events, but reasons for participation in those activities were to find comfort and peace and connect with God. Krause et al. (2019) concluded that people who get supported spiritually by fellow church members are more likely to integrate more compassion and forgiveness into their lives, and those who help others experience a greater perception of meaning in life.

We observed that individuals who believed in a higher being were less likely to feel hopeless. Dolcos et al. (2021) also showed a negative correlation between religious coping and anxiety traits as well as depression scores, meaning that patients who used religion for coping also experienced less anxiety and depressive symptoms. Gusar et al. (2021) demonstrated that generally lower psychological states and higher levels of anxiety and depression were found in non-religious ophthalmologic patients who opposed religion.

In our study, international participants who were generally happy in life, found comfort in prayer when they experienced moments of fear of losing their vision, making prayer a valuable tool in coping with the anxiety of facing a disease that might lead to blindness. Sharp (2010) showed that people who used prayer as a resource for individual emotional management by surrendering anger maintained their self-esteem through a positive self-assessment in prayer and gained a new view that was less threatening on their condition. Additionally, international participants in our study were generally happy in life when they found comfort and strength in their faith, using it to cope with AMD on a daily basis, compared to patients who reported currently struggling more with their faith or experiencing a fluctuation between faith as a source of strength and faith as more of a struggle, and patients who reported to not believe. The finding was similar for international participants who reported feeling generally hopeful in their life.

We found interesting insights when we examined the correlation between happiness and religion in different countries. Stavrova et al. (2013) observed that religiosity had a stronger positive influence on happiness and life gratification in countries where religiosity represented a social norm. In their study, they looked at 64 countries between 2005 and 2008 and examined the social norm of religiosity in these countries. Descriptive statistics from their study pertaining to Romania, Germany, and the USA (the countries we analyzed for correlation between happiness and religion) are presented in Table 12. From these statistics, it is evident that Romania has a higher norm of religiosity and Germany has the lowest, while the USA has the most diversity in terms of religious denominations; the diversity in religious denominations is higher when the country’s score of religious fractionalization is high (Stavrova et al., 2013).

**TABLE 12:** Country-wise descriptive statistics of the social norm of religiosity. Source: Stavrova et al., 2013.

Country	*N*	Country’s norm of religiosity (a)	Religious fractionalization (b)	Dominant religion
Romania	2,963	1.11	0.24	Orthodox
Germany	3,640	−1.30	0.66	Catholic
USA	1,175	0.27	0.82	Other Christian

(a) z-standardized values. Higher values indicate a stronger norm of religiosity. (b) Higher values indicate stronger religious heterogeneity.

In our study, we found that AMD patients from the USA generally reported “feel cared for” in their lives, compared to participants from Romania or Germany, and patients from the latter were the ones who least felt cared for. When reviewing the findings of Stavrova et al., as seen in Table 12, we found that Germany had the least strong norm of religiosity among the three countries we analyzed. Though we had not specified what feeling “cared for” meant, we included “feeling cared for” in the section where we generally asked about the emotional state of the patients. “Feeling cared for” could be understood by the patients as support from family, having someone who takes care of them, or how cared for they felt by their physician during treatments. Multiple factors may have contributed to the patients feeling cared for in their lives. A study by Gans et al. (2009) revealed that filial piety and the guardianship of parents were connected to religiosity across five different national contexts.

According to our results, the less a person felt cared for, the more likely they will attend religious events. On the other hand, this could also be interpreted that patients who attended religious events, might have higher expectations of care from their family, compared to non-religious patients, due to the examples of care toward parents and elders given in sacred scriptures.

Rote et al. (2013) found that religious participation was linked to a higher level of social integration. Dunbar (2021) study highlighted that religious attendance was connected to a sense of bonding with the members of a congregation. Social engagement could be a factor in making patients “feel cared for” in their lives, thus encouraging them to participate in religious activities.

International patients in our studies were reported to be generally happy in their lives when they believed that God could make “a blessing out of their disease.” The same applied to those who were generally “feeling hopeful” in their lives. These findings could not be determined in the Romanian group. On the other hand, Romanian patients were found to generally “feel down in their lives” when they also believed that “the disease is a punishment.” The same applied to those who generally felt hopeless in their lives. However, it must be mentioned that only a minority of patients chose the “disease is a punishment” response option in the Romanian group.

In their study of ophthalmologic patients, Magyar-Russell et al. (2008) concluded that “positive” religious conceptualizations of the diseases are much higher than the “negative” ones. However, careful attention should be paid since negative religious appraisals can favor the development of depression (Williams and Sternthal, 2007; Holt et al., 2018) and lead to anxiety and negative personality traits (Koenig, 2012).

In the Romanian group, patients who believed in a higher power also believed that “God can heal the disease,” and patients who participated in religious activities were of the view that “God can make a blessing out of the disease,” thus having a positive religious appraisal.

Interestingly, Romanian patients who required assistance in their daily activities expressed a desire to talk about God with the medical staff. In fact, Romanian and international patients who believed in a higher power wanted to have this conversation with the medical team. Our finding is supported by Magyar-Russell et al. (2008) who examined religious and spiritual beliefs in ophthalmologic patients and found that 23.4% of patients preferred to talk to their physician about the role they believed God played in their ophthalmologic disease. Earlier, King and Bushwick (1994) investigated the desire of patients to talk about their religious beliefs with their physicians through a cross-sectional survey conducted at two different hospitals in the USA with 203 in-patients who were hospitalized for three or more days. The study found that 77% of patients wanted their physicians to consider their spiritual needs, 37% wanted their physicians to discuss spiritual topics more frequently, and 48% wished their physicians to pray with them. The study also noted that 68% of patients reported that their physicians never talked about faith with them.

Ehman et al. (1999) surveyed 177 patients at a pulmonary outpatient practice at the hospital of the University of Pennsylvania to find out if patients wanted their physicians to discuss with them their spiritual or religious beliefs if they became gravely ill. In the survey, 45% of patients agreed with the statement that their religious or spiritual beliefs would influence their medical decision in case they became gravely ill, and 94% would want their physician to ask them about it if they were severely sick. Even though 54% of the patients did not agree that their religious or spiritual beliefs would influence their medical decision in such a case, yet they would prefer the physician to ask them about such a belief. Regardless, 66% of the patients agreed that if their physician would discuss religious or spiritual beliefs with them, it would strengthen their trust in their attending healthcare provider.

In Kansas, Texas (USA), a similar study was undertaken with 80 patients at a university-based family practice center, and it obtained similar results. The study found that if patients participated in religious activities at least once a month, they were open to the physician discussing with them their religion and personal faith, and they welcomed any referral to pastoral professionals in case of spiritual issues (Daaleman and Nease, 1994). A recent study on 236 cancer survivors also concluded that for patients who saw themselves as religious, integrating their religious and spiritual needs into their cancer care regimen was important, although it has to be mentioned that 68% of patients disregarded their religious or spiritual belonging and did not want to discuss spiritual and religious topics with their physician (Palmer Kelly et al., 2021). In yet another study, Williams et al. (2011) found that 41% of hospitalized patients in their research group would like to have a conversation about religion and spirituality but only half the patients who desired such conversations actually had them. Another interesting finding was reported by Cipriano-Steffens et al. (2020) in their study. According to them, providing patients the opportunity to talk about spirituality and their strategy to cope with cancer filled the patients with a sense of peace and had a lowering effect on stress; having someone listen to their way of coping was a form of coping mechanism in itself.

Several of these studies confirm the findings from our study that Romanian and international patients who considered themselves religious expressed an increase in their interest in spiritual and religious topics with the onset of AMD. The Romanian patients who disclosed being more interested in spiritual and religious topics through their disease also expressed an interest to have conversations about God with their health professionals.

The findings of our study are also echoed in the study by Gusar et al. (2021) which described how having AMD and depression led to a significant rise in religiousness, which was not the case for patients with other ophthalmological conditions such as glaucoma and diabetic retinopathy. Furthermore, the data obtained from the Romanian group suggests that patients who generally felt fearful in life had a statistically significant higher mean of agreeing to the statement that having AMD made them more interested in spiritual and religious topics, compared to those who only partially or did not agree at all. The fear of losing one’s vision and the uncertain and challenging life circumstances that accompany such a loss might inspire a person to seek meaning beyond what can be seen with the physical eye.

## 4.1. Limitations

Several limitations should be taken into consideration. First, we had a small sample size. The international group consisted of patients from different countries. Some countries such as the Philippines or others had only one participant. A limited geographic area with limited cultures was included in our study.

We also included Germany in the international group, which might be considered a more secular country in comparison to the USA or others. Therefore, in future research, it would be interesting to compare more secular societies with more conservative ones.

Second, the survey for the international group was administered online, so only those who had access to the Internet could participate. Meanwhile, the Romanian patients received the survey at the clinic and could fill it out on-site with assistance, if needed, or at home.

Third, patients with visual impairment could not always fill out the survey by themselves, as some of them needed assistance. In our survey, we enquired about their emotional state. If they received assistance in filling out the survey, we cannot know how comfortable patients felt answering those questions sincerely.

Fourth, some patients from the international group who reported having the dry form of AMD also answered that they had received an intraocular injection in the past. It is possible that patients were not fully aware of which type of AMD they had, and at which stage of the disease they were currently in since intraocular injections are only given in the exudative (wet) AMD.

Fifth, religious coping was only assessed in the form of prayers, meditation, or participation in religious activities. Non-religious coping mechanisms should be examined too. It should also be taken into consideration that not all spiritual people are religious.

Sixth, the definition and differentiation of “religion” and “spirituality” are challenging. More precise questions should be added. A multidimensional approach is needed to understand the pathways of how spirituality and religion influence the health of an individual.

More precise scores should be used for the examination of the “quality of life” and “emotional state.”

### 4.2. Implications for future research

For further research, the inclusion of secular countries and a comparison with more conservative countries would be interesting. In addition, it would be beneficial to have a study with a wider research scope that includes persons with visual impairment drawn from diverse religious beliefs, including atheists, and a bigger sample size.

It would be also of value to assess spirituality and religiosity at the beginning of the AMD diagnosis, and then again in later stages after disease progression, to examine the perspective and determine changes in spirituality and religiosity over time.

Moreover, the implementation of clinical data such as visual acuity and OCTs of patients into survey analysis would be very useful.

## 5. Conclusion

Our study established that spirituality and religion are important factors that facilitate patients’ ability to cope with a progressive degenerative disease like AMD. Patients who are religious are more at peace with having AMD. Practices that contribute to peace that comes with the acceptance of the disease are regular prayer or meditation, as data from the international group revealed. Moreover, prayer helped patients to deal with the fear of vision loss and thus provided comfort and strength.

Spirituality and religion are important components that promote a healthier and happier emotional state and mental wellbeing. By believing that death is not the end, patients felt more hopeful, which helped in their adjustment to a health condition that otherwise appeared hopeless.

The positive effect of religion and spirituality on emotional wellbeing also depends on what image the individual portrays of God, although the majority have a benevolent image of the higher being and God’s role in the illness. A positive religious appraisal provides a better psychological adjustment to critical life events.

On whether patients desired to talk about God with their medical staff, we concluded that about half of the Romanian patients agreed with that, while in the international group, this desire was less. The profile of a patient who desires to talk about God with their healthcare professional could be a believer in a higher power, someone who prays/meditates often, participates in religious activities, is worried about the loss of vision, or needs assistance in daily life. In treatment protocols, adopting an interdisciplinary and multidimensional approach to medical health professionals, mental health workers, and pastoral professionals would be of great value.

## Data availability statement

The raw data supporting the conclusions of this article will be made available by the authors, without undue reservation.

## Ethics statement

The studies involving human participants were reviewed and approved by the Iuliu Hatieganu University–Ethics Committee. The patients/participants provided their written informed consent to participate in this study.

## Author contributions

CS and OS contributed to the conception and design of the study and revised the final version of the manuscript. LS contributed to data collection and manuscript revision. HMM contributed to statistical analyses and manuscript revision. All authors contributed to the article and approved the submitted version.

## References

[B1] Alma M. A. Van der Mei S. F. Feitsma W. N. Groothoff J. W. Van Tilburg T. G. Suurmeijer T. P. (2011). Loneliness and self-management abilities in the visually impaired elderly. *J. Aging Health* 23 843–861. 10.1177/0898264311399758 21398572

[B2] Barber C. (2012). Spirituality and religion: a brief definition. *Br. J. Healthcare Assistants* 6 378–381. 10.12968/bjha.2012.6.8.378

[B3] Benjamins M. R. (2006). Religious influences on trust in physicians and the health care system. *Int. J. Psychiatry Med.* 36 69–83. 10.2190/EKJ2-BCCT-8LT4-K01W 16927579

[B4] Büssing A. (2021). The spiritual needs questionnaire in research and clinical application: a summary of findings. *J. Religion Health* 60 3732–3748. 10.1007/s10943-021-01421-4 PMC8484079 34491481

[B5] Büssing A. Franczak K. Surzykiewicz J. (2014). Frequency of spiritual/religious practices in polish patients with chronic diseases: validation of the polish version of the SpREUK-P questionnaire. *Religions* 5 459–476. 10.3390/rel5020459 25344880

[B6] Casten R. J. Rovner B. W. (2013). Update on depression and age-related macular degeneration. *Curr. Opin. Ophthalmol.* 24 239–243. 10.1097/ICU.0b013e32835f8e55 23429599 PMC5903583

[B7] Cipriano-Steffens T. M. Carilli T. Hlubocky F. Quinn M. Fitchett G. Polite B. (2020). “Let Go, Let God”: a qualitative study exploring cancer patients’ spirituality and its place in the medical setting. *J. Religion Health* 59 2341–2363. 10.1007/s10943-019-00942-3 31705446

[B8] Daaleman T. P. Nease D. E. Jr. (1994). Patient attitudes regarding physician inquiry into spiritual and religious issues. *J. Family Practice* 39 564–568. 7798860

[B9] De Sousa Peixoto R. Krstic L. Hill S. Foss A. (2021). Predicting quality of life in AMD patients—insights on the new NICE classification and on a bolt-on vision dimension for the EQ-5D. *Eye* 35 3333–3341. 10.1038/s41433-021-01414-3 33526850 PMC8602239

[B10] Demmin D. L. Silverstein S. M. (2020). Visual impairment and mental health: unmet needs and treatment options. *Clin. Ophthalmol.* 14 4229–4251. 10.2147/OPTH.S258783 33299297 PMC7721280

[B11] Dolcos F. Hohl K. Hu Y. Dolcos S. (2021). Religiosity and resilience: cognitive reappraisal and coping self-efficacy mediate the link between religious coping and well-being. *J. Religion Health* 60 2892–2905. 10.1007/s10943-020-01160-y 33415601 PMC7790337

[B12] Dunbar R. I. M. (2021). Religiosity and religious attendance as factors in wellbeing and social engagement. *Religion Brain Behav.* 11 17–26. 10.1080/2153599X.2020.1712618

[B13] Ehman J. W. Ott B. B. Short T. H. Ciampa R. C. Hansen-Flaschen J. (1999). Do patients want physicians to inquire about their spiritual or religious beliefs if they become gravely Ill? *Arch. Int. Med.* 159 1803–1806. 10.1001/archinte.159.15.1803 10448785

[B14] Ellison C. G. Boardman J. D. Williams D. R. Jackson J. S. (2001). Jackson, religious involvement, stress, and mental health: findings from the 1995 Detroit area study. *Soc. Forces* 80 215–249. 10.1353/sof.2001.0063 34409987

[B15] Finger R. P. Fenwick E. Marella M. Dirani M. Holz F. G. Peggy P. C. (2011). The impact of vision impairment on vision-specific quality of life in germany. *Invest. Ophthalmol. Vis. Sci.* 52 3613–3619. 10.1167/iovs.10-7127 21357395

[B16] Gans D. Silverstein M. Lowenstein A. (2009). Do religious children care more and provide more care for older parents? a study of filial norms and behaviors across five nations. *J. Comp. Family Stud.* 40 187–201. 10.3138/jcfs.40.2.187 PMC4507809 26203200

[B17] Garssen B. Uwland-Sikkema N. F. Visser A. (2015). How spirituality helps cancer patients with the adjustment to their disease. *J. Religion Health* 54 1249–1265. 10.1007/s10943-014-9864-9 24748130

[B18] Gusar I. Čanović S. Ljubičić M. Šare S. Perić S. Caktaš I. L. (2021). Religiousness, anxiety and depression in patients with glaucoma, age-related macular degeneration and diabetic retinopathy. *Psychiatria Danubina* 33 (Suppl. 4), 965–973. 35026829

[B19] Holt C. L. Roth D. L. Huang J. Clark E. M. (2018). Role of religious social support in longitudinal relationships between religiosity and health-related outcomes in African Americans. *J. Behav. Med.* 41 62–73. 10.1007/s10865-017-9877-4 28776192 PMC5766361

[B20] Hvidtjørn D. Hjelmborg J. Skytthe A. Christensen K. Hvidt N. C. (2014). Religiousness and religious coping in a secular society: the gender perspective. *J. Religion Health* 53 1329–1341. 10.1007/s10943-013-9724-z 23625173 PMC4226847

[B21] Hyman C. Handal P. J. (2006). Definitions and evaluation of religion and spirituality items by religious professionals: a pilot study. *J. Religion Health* 45 264–282. 10.1007/s10943-006-9015-z

[B22] Keinki C. Meyer H. Bozkurt G. Müller N. Römelt J. Müller U. A. (2022). Salvation expectations of patients of medicine, complementary and alternative medicine and religion. *J. Religion Health* 61 601–615. 10.1007/s10943-020-01074-9 32948977 PMC8837522

[B23] Kim S. Park S. J. Byun S. J. Park K. H. Suh H. S. (2019). Incremental economic burden associated with exudative age-related macular degeneration: a population-based study. *BMC Health Serv. Res.* 19:828. 10.1186/s12913-019-4666-0 31718629 PMC6852978

[B24] King D. E. Bushwick B. (1994). Beliefs and attitudes of hospital inpatients about faith healing and prayer. *J. Family Practice* 39 349–352. 7931113

[B25] Koenig H. G. (2004). Religion, spirituality, and medicine: research findings and implications for clinical practice. *Southern Med. J.* 97 1194–1200. 10.1097/01.SMJ.0000146489.21837.CE 15646757

[B26] Koenig H. G. (2009). Research on religion, spirituality, and mental health: a review. *Can. J. Psychiatry* 54 283–291. 19497160 10.1177/070674370905400502

[B27] Koenig H. G. (2012). Religion, spirituality, and health: the research and clinical implications. *ISRN Psychiatry* 2012:278730. 10.5402/2012/278730 23762764 PMC3671693

[B28] Krause N. Hill P. C. Ironson G. (2019). Evaluating the relationships among religion, social virtues, and meaning in life. *Arch. Psychol. Religion* 41 53–70. 10.1177/0084672419839797

[B29] Li J. Q. Welchowski T. Schmid M. Mauschitz M. M. Holz F. G. Finger R. P. (2020). Prevalence and incidence of age-related macular degeneration in europe: a systematic review and meta-analysis. *Br. J. Ophthalmol.* 104 1077–1084. 10.1136/bjophthalmol-2019-314422 31712255

[B30] Li Y. I. Woodberry R. Liu H. Guo G. (2020). Why are women more religious than men? do risk preferences and genetic risk predispositions explain the gender gap? *J. Sci. Study Religion* 59 289–310. 10.1111/jssr.12657 33071306 PMC7566885

[B31] Magyar-Russell G. Fosarelli P. Taylor H. Finkelstein D. (2008). Ophthalmology patients’ religious and spiritual beliefs: an opportunity to build trust in the patient-physician relationship. *Arch. Ophthalmol.* 126 1262–1265. 10.1001/archopht.126.9.1262 18779488

[B32] Marzorati C. Pravettoni G. (2017). Value as the key concept in the health care system: how it has influenced medical practice and clinical decision-making processes. *J. Multidisciplinary Healthcare* 10 101–106. 10.2147/JMDH.S122383 28356752 PMC5367583

[B33] Naghi J. J. Philip K. J. Phan A. Cleenewerck L. Schwarz E. R. (2012). The effects of spirituality and religion on outcomes in patients with chronic heart failure. *J. Religion Health* 51 1124–1136. 10.1007/s10943-010-9419-7 23304705

[B34] Palmer Kelly E. Paredes A. Z. DiFilippo S. Hyer M. Tsilimigras D. I. Rice D. (2021). The religious/spiritual beliefs and needs of cancer survivors who underwent cancer-directed surgery. *Palliative Supportive Care* 19 175–181. 10.1017/S1478951520000772 32854807

[B35] Pargament K. I. (1997). *The Psychology of Religion and Coping. Theory, Research, Practice.* New York, NY: Guilford Press.

[B36] Popescu M. L. Boisjoly H. Schmaltz H. Kergoat M. J. Rousseau J. Moghadaszadeh S. (2012). Explaining the relationship between three eye diseases and depressive symptoms in older adults. *Invest. Ophthalmol. Visual Sci.* 53 2308–2313. 22427589 10.1167/iovs.11-9330

[B37] Rassoulian A. Gaiger A. Loeffler-Stastka H. (2021). Gender differences in psychosocial, religious, and spiritual aspects in coping: a cross-sectional study with Cancer patients. *Women’s Health Rep.* 2 464–472. 10.1089/whr.2021.0012 PMC8617579 34841392

[B38] Reid-Arndt S. A. Smith M. L. Yoon D. L. Johnstone B. (2011). Gender differences in spiritual experiences, religious practices, and congregational support for individuals with significant health conditions. *J. Religion Disabil. Health* 15 175–196. 10.1080/15228967.2011.566792

[B39] Riklikienė O. Tomkevičiūtė J. Spirgienė L. Valiulienė Ž Büssing A. (2020). Spiritual needs and their association with indicators of quality of life among non-terminally ill cancer patients: cross-sectional survey. *Eur. J. Oncol. Nursing* 44:101681. 10.1016/j.ejon.2019.101681 31816507

[B40] Rote S. Hill T. D. Ellison C. G. (2013). Religious attendance and loneliness in later life. *Gerontologist* 53 39–50. 10.1093/geront/gns063 22555887 PMC3551208

[B41] Rovner B. W. Casten R. J. Leiby B. E. Tasman W. S. (2009). Activity loss is associated with cognitive decline in age-related macular degeneration. *Alzheimer’s Dement.* 5 12–17. 10.1016/j.jalz.2008.06.001 19118805 PMC3586705

[B42] Sharp S. (2010). How does prayer help manage emotions? *Soc. Psychol. Quarterly* 73 417–437. 10.1177/0190272510389129

[B43] Shattuck E. C. Muehlenbein M. P. (2020). Religiosity/spirituality and physiological markers of health. *J. Religion Health* 59 1035–1054. 10.1007/s10943-018-0663-6 29978269

[B44] Sherman A. C. Simonton-Atchley S. O’Brien C. E. Campbell D. Reddy R. M. Guinee B. (2021). Associations between religious/spiritual coping and depression among adults with cystic fibrosis: a 12-Month longitudinal study. *J. Religion Health* 60 2646–2661. 10.1007/s10943-021-01185-x 33575892

[B45] Stavrova O. Fetchenhauer D. Schlösser T. (2013). Why are religious people happy? the effect of the social norm of religiosity across countries. *Soc. Sci. Res.* 42 90–105. 10.1016/j.ssresearch.2012.07.002 23146600

[B46] Taylor D. J. Jones L. Binns A. M. Crabb D. P. (2020). ’You’ve got dry macular degeneration, end of story’: a qualitative study into the experience of living with non-neovascular age-related macular degeneration. *Eye* 34 461–473. 10.1038/s41433-019-0445-8 31118490 PMC7042256

[B47] Waern M. Rubenowitz E. Runeson B. Skoog I. Wilhelmson K. Allebeck P. (2002). Burden of illness and suicide in elderly people: case-control study. *BMJ* 324:1355. 10.1136/bmj.324.7350.1355 12052799 PMC115206

[B48] Wahl H. W. Schilling O. Oswald F. Heyl V. (1999). Psychosocial consequences of age-related visual impairment: comparison with mobility-impaired older adults and long-term outcome. *J. Gerontol. B. Psychol. Sci. Soc. Sci.* 54 304–316. 10.1093/geronb/54b.5.p304 10542823

[B49] Wang C. W. Chan C. L. Ng S. M. Ho A. H. (2008). The impact of spirituality on health-related quality of life among Chinese older adults with vision impairment. *Aging Mental Health* 12 267–275. 10.1080/13607860801951903 18389408

[B50] Williams D. R. Sternthal M. J. (2007). Spirituality, religion and health: evidence and research directions. *Med. J. Australia* 186 S47–S50. 10.5694/j.1326-5377.2007.tb01040.x 17516883

[B51] Williams J. A. Meltzer D. Arora V. Chung G. Curlin F. A. (2011). Attention to inpatients’ religious and spiritual concerns: predictors and association with patient satisfaction. *J. General Int. Med.* 26 1265–1271. 10.1007/s11606-011-1781-y 21720904 PMC3208457

[B52] World Health Organization (2022). *Vision Impairment and Blindness.* Geneva: WHO.

[B53] Yampolsky M. Wittich W. Webb G. Overbury O. (2008). The role of spirituality in coping with visual impairment. *J. Vis. Impairm. Blindness* 102 28–39. 10.1177/0145482X0810200104

[B54] Yanguas J. Pinazo-Henandis S. Tarazona-Santabalbina F. J. (2018). The complexity of loneliness. *Acta Biomed.* 89 302–314. 10.23750/abm.v89i2.7404 29957768 PMC6179015

